# Editorial: Frontiers in dental medicine: highlights in systems integration 2021/22

**DOI:** 10.3389/fdmed.2023.1208248

**Published:** 2023-06-02

**Authors:** Emily Chu, Francisco H. Nociti

**Affiliations:** ^1^ Department of General Dentistry, University of Maryland School of Dentistry, Baltimore, MD, United States; ^2^ Cellular and Molecular Biology Research Group, American Dental Association, Science and Research Institute (ADARSI), Institute of Technology and Research, Gaithersburg, MD, United States

**Keywords:** radiology, orthodontics & dentofacial orthopedics, diabetes—quality of life, alveolar bone, obstructive sleep

**Editorial on the Research Topic** Frontiers in dental medicine: highlights in systems integration 2021/22

## Introduction

Frontiers in Dental Medicine (FDMED) was launched and presented a vision to set the stage for advancing an integrative and multidisciplinary approach at several levels, ranging from basic science, clinical practice, to health policy decision making. In July 2022, the first Research Topic on the Highlights of Systems Integration: 2021 was released with two articles that discussed about the potential of artificial intelligence (AI) to assist clinicians in improving data analysis, and therefore, demonstrating its potential to support the implementation of evidence-based dentistry.

As we constantly and continuously keep working toward making FDMED one of the most cited journal in the dental-oral-craniofacial field, we are thrilled to concluded one more Research Topic of seven peer-reviewed articles.

Pae and Harper determined whether hyoid bone had the ability to predict the effectiveness of an anterior mandibular positioning appliance in individuals diagnosed with obstructive sleep apnea (OSA). The authors found that the appliances effectiveness was dependent on the mode of hyoid elevations, likely resulting from muscle responsiveness in patients with AMP use. Their observations suggest that hyoid elevation may be a useful marker to segregate patients highly responsive to AMP from those not-so-responsive.

Shimpi et al. performed a statewide survey among Wisconsin-based dental providers to determine their current knowledgeability and attitudes when managing patients with diabetes/prediabetes in the dental setting. The authors aimed at exploring perceptions on feasibility, value, barriers, and current status of integrated care model (ICM) adoption by dental providers. A survey was mailed to all licensed dentists and dental hygienists practicing in Wisconsin, and the authors suggested a need for educational curricula reform.

Finally, Macrì et al. retrospectively assessed the prevalence of an important anomaly of the atlas (C1 vertebra) called ponticulus posticus (PP), displaying a complete or partial bone bridge that has the potential to transform the groove of the vertebral artery (VA) into a canal (arcuate foramen). The authors used morphological assessments and determined a potential association with other dentoskeletal anomalies. They concluded that bilateral partial variant and bilateral complete variant are the most represented morphotypes in all the age groups, with the MCI featuring a positive association with both, PP and SB.

We hope this Research Topic will help both, researchers and clinicians, to adopt the latest knowledge into their daily work indicating that a relevant progress has been made towards utilizing a systems-based approach to support the practice of evidence-based dentistry and to improve dental treatment outcomes.

